# Distance decay and persistent health care disparities in South Africa

**DOI:** 10.1186/s12913-014-0541-1

**Published:** 2014-11-04

**Authors:** Zoë M McLaren, Cally Ardington, Murray Leibbrandt

**Affiliations:** Department of Health Management and Policy, School of Public Health, University of Michigan, 1415 Washington Heights, Ann Arbor, MI 48109 USA; Southern Africa Labour and Development Research Unit (SALDRU), University of Cape Town, Private Bag X3, Rondebosch, 7701 South Africa; NRF Research Chair in Poverty and Inequality Research, Director of SALDRU, University of Cape Town, Private Bag X3, Rondebosch, 7701 South Africa

**Keywords:** Health care utilization, Inequality, South Africa, Distance decay

## Abstract

**Background:**

Access to health care is a particular concern given the important role of poor access in perpetuating poverty and inequality. South Africa’s apartheid history leaves large racial disparities in access despite post-apartheid health policy to increase the number of health facilities, even in remote rural areas. However, even when health services are provided free of charge, monetary and time costs of travel to a local clinic may pose a significant barrier for vulnerable segments of the population, leading to overall poorer health.

**Methods:**

Using newly available health care utilization data from the first nationally representative panel survey in South Africa, together with administrative geographic data from the Department of Health, we use graphical and multivariate regression analysis to investigate the role of distance to the nearest facility on the likelihood of having a health consultation or an attended birth.

**Results:**

Ninety percent of South Africans live within 7 km of the nearest public clinic, and two-thirds live less than 2 km away. However, 14% of Black African adults live more than 5 km from the nearest facility, compared to only 4% of Whites, and they are 16 percentage points less likely to report a recent health consultation (p < 0.01) and 47 percentage points less likely to use private facilities (p < 0.01). Respondents in the poorest income quintiles live 0.5 to 0.75 km further from the nearest health facility (p < 0.01). Racial differentials in the likelihood of having a health consultation or an attended birth persist even after controlling for confounders.

**Conclusions:**

Our results have two policy implications: minimizing the distance that poor South Africans must travel to obtain health care and improving the quality of care provided in poorer areas will reduce inequality. Much has been done to redress disparities in South Africa since the end of apartheid but progress is still needed to achieve equity in health care access.

## Introduction

Inequality in access to health care is an important concern for health policy in developing countries. Because health status influences human capital acquisition, economic status and the inter-generational transmission of socio-economic status, access to care plays a role in determining and reinforcing other measures of inequality [1,2]. These factors are particularly important in South Africa because the legacy of apartheid leaves non-whites in remote areas, which are potentially underserved [3,4]. Public health services are often subsidized as a means to promote equitable access. In post-apartheid South Africa, the government has emphasized equity and made access to clinics the centerpiece of primary health care [3,5,6]. This makes it important to understand which members of the population actually benefit from these services and who is being left behind [1,7].

Even when health services are provided free of charge, monetary and time costs of travel to a local clinic represent the price of access to health care. These costs may pose a significant barrier for vulnerable segments of the population, leading to overall poorer health. Twenty years after the end of apartheid, residential location remains largely racially defined, which can exacerbate barriers if health facilities are located far from non-White neighborhoods [8]. Travel costs in South Africa are particularly high relative to other developing countries in Africa and elsewhere, which means that small differences in distance can translate into large differences in access [9].

In this study we investigate the role of distance to the nearest health facility in driving patterns of health care utilization in South Africa. We make use of new data from the National Income Dynamics Study (NIDS) [10]. We start with a descriptive analysis of differences in proximity to health facilities and patterns of care seeking behavior by race and income at the national level to contextualize the inequality in utilization of health care. We use multivariate regression with a rich set of control variables to investigate the relationship between proximity and utilization of health care.

Despite the importance of patterns of health service utilization in determining health care financing and delivery, there is a limited literature on the role of travel costs in developing countries. The major challenge is obtaining reliable measures of travel time and monetary travel cost to access health services. This requires information on the precise location of the household and the health facility, network of roads, availability of and waiting time for public transportation, and reliability of self-reported data. In South Africa, studies have been conducted in a Demographic Surveillance Site in rural KwaZulu-Natal; however, it is unclear whether these estimates can be generalized to the rest of the country [11,12]. Indeed, there are few large-scale studies of this sort in developing countries and the existing nationally representative studies rely on self-reported estimates of distance or time to the nearest clinic [3,6].

In sum, our contribution here is two-fold. First, we assemble and verify geographic coordinates for all health facilities in South Africa, drawing on partial lists from several sources. We are the first to use nationally representative facility- use data from NIDS. These data, in combination with the information in the NIDS survey on individuals and their households, provide a rich set of control variables that the literature has highlighted as important in determining health care access and utilization. Second, we conduct our analysis in a context where high racial, gender and income inequality as well as substantial travel costs create salient frictions in health care access and utilization. We contribute to a limited literature where the need for policy guidance is great.

## Background

Distance decay in utilization of health services has been documented in many contexts, including utilization of diarrhea clinics in Bangladesh [13], care for malaria and acute respiratory infections in Papua New Guinea [14], health facilities in rural Nigeria [15], health services in rural Ghana [16], care for young children [17-19], and health facilities by the rural elderly in the United States [20]. There are differences in the rate of distance decay by gender and age [14]; income, education and cost of services [16]; socio-demographic factors and village characteristics [15]; and access to public or private transportation [20]. Education levels and employment status influence women’s health-seeking behavior in Ethiopia [21] as does freedom of movement in Muslim North India [22].

Tanser et al. [11] find evidence of distance decay in primary health care utilization in South Africa. Even in the case of take-up of life-saving anti-retroviral therapy for AIDS, there is a strong negative association with distance to the nearest facility: individuals 5kms from the nearest clinic are only half as likely to access ART as those living next-door to the facility [12]. These results are demonstrative of the importance of distance in mediating care-seeking behavior. If distance reduces take-up of a life-saving treatment regime, it surely reduces take-up of less essential care.

## Data

Our data come from the first wave of the NIDS, the first nationally representative panel survey in South Africa. The NIDS data include information on income, expenditure, household composition, fertility, mortality, human capital formation, health and social capital [23]. The first wave, conducted in 2008, surveyed and took biometric measurements of every individual in 7,305 households – a total of 15,634 adults and 9,408 children under 15. Important for this study is that the data set contains an especially rich set of individual, household and community level characteristics. Each adult respondent that reported consulting someone about their health in the past year was asked for the name and location of the health facility where the consultation took place. During fieldwork, GPS co-ordinates of the household were taken using handheld GPS units and then transcribed onto the paper questionnaire. This is the first study to use the confidential data on the location of clinic attended together with the location of respondent’s household.

Our data on health facilities were shared by five public sources (National Department of Health (DOH), Western Cape DOH, KwaZulu-Natal DOH, Human Sciences Research Council and National Health Laboratory Service) and purchased from two private sources (MedPages and AfriGIS), which we combined to create a master list of all facilities. The data include facility name, facility type (e.g. clinic, hospital, etc.), health district and geographic coordinates. We compared partially overlapping lists to verify names and geographic coordinates of each facility.

## Methods

We calculated the exact distance between households and their nearest public clinic using geographic positioning system (GPS) coordinates. We follow Tanser et al. [24] and use Euclidean distance as a measure of distance traveled. In South Africa, reliable geocoding is not available to calculate travel time for respondents throughout the country. Rosero-Bixby [25] found the correlation between Euclidean distances and travel times to be high, but not perfect. In the absence of geocoding, Euclidean distance provides a good measure of travel costs.

Using this variable, we generate descriptive means of the sample, and perform multivariate regression analysis to investigate the determinants of proximity to public health facilities. We then perform our main analyses using multivariate regression analysis to investigate distance decay in health care utilization, controlling for several individual and household characteristics.

For ease of interpretation, we estimate linear regression models and report regression coefficients (marginal effects). All substantive findings are robust to the use of logistic regression (results not shown but available on request). Our estimating equation is as follows:

$$ {Y}_{ij}=\beta {D}_j+\phi '{X}_{ij}+{\varepsilon}_{ij}, $$

where *Y*_*ij*_ is a binary indicator for the health care utilization outcome (health consultation in the last year for adults, skilled attendant at birth for children) for individual *i* in household *j*, *D*_*j*_ is a measure of distance to the nearest public health facility, and *X*_*ij*_ is a rich set of individual and household-level control variables that have been identified by the literature as important determinants of health care access and utilization. Regression tables report results from multiple regression specifications that include a combination of controls for race, sex, age, income and household composition. Some specifications with adult samples include controls for education level and self-reported health status. Other specifications include interaction terms between race and the distance to the nearest clinic to capture any racial differences in distance decay. We remove from the estimation sample of adult respondents aged 18 and older the small number of respondents who indicate that they have recently moved (i.e. since 2006). Similarly, we excluded any children who have moved since birth from our sample of children aged 5 years and under. All descriptive statistics and regressions are weighted using the post-stratification weights provided by NIDS. Standard errors are clustered at the primary sampling unit level and are robust to heteroskedasticity. Ethics approval for this work was granted by the University of Cape Town Commerce Faculty Ethics in Research Committee.

## Results

**Figure 1:**
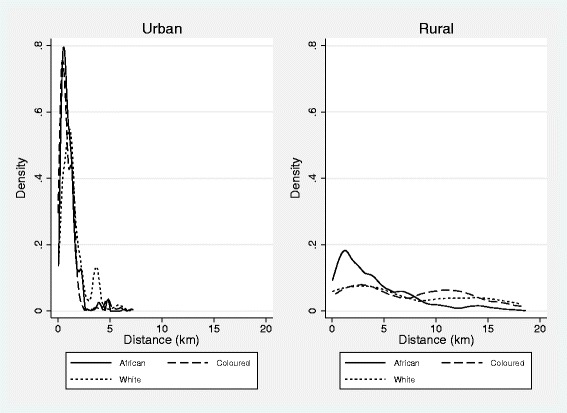
**Distance to closest clinic by rural/urban location and population group (race).** Authors’ calculation using NIDS data and post-stratification weights. For the 2% of households that are multi-racial, race group is defined as the majority race of household residents.

Just under half of African households (49%) are located in rural areas as opposed to 32% of Coloured and 16% of White households. This is associated with racial disparities in proximity to health facilities shown in Table 1 that summarizes the distance of the household from the nearest public health facility by the majority race group in the household. Fourteen percent of Black African households live more than 5 km from the nearest public health facility, in contrast to only 8% of Coloureds and 4% of Whites. Figure 1 shows that these stark racial differences are driven by differences between rather than within rural and urban areas. Indeed, after conditioning on urban or rural residence, white households tend to be located furthest away from public health facilities.

**Table 1:** **Distance to public clinics by population group (race) and per capita income quintile for South African households**

		**(1)**	**(2)**	**(3)**	**(4)**	**(5)**
		**Distance from household to closest public clinic**				
		**N**	**Distance (km)**	**<2 km**	**2-5 km**	**>5 km**
Race:						
	Black African	5,611	2.61	0.669	0.187	0.144
	Coloured	1,004	2.04***	0.884***	0.042***	0.075***
	White	551	1.93***	0.674	0.287***	0.039***
Per capita income quintile:						
	1	1,620	3.01	0.578	0.222	0.200
	2	1,793	2.68**	0.629***	0.223	0.148***
	3	1,635	2.48***	0.714***	0.160***	0.126***
	4	1,226	2.21***	0.791***	0.117***	0.092***
	5	927	1.95***	0.735***	0.214	0.052***

Authors’ calculation using NIDS and health facility data and post-stratification weights. For the 2% of households that are multi-racial, race group is defined as the majority race of household residents. The number of households differs across per capita income quintiles as households were assigned to quintiles after taking post-stratification weights into account. Asterisks in the top panel of columns 2 to 5 indicate whether White and Coloured means are statistically significantly different from Black African means at the 1% (***) and 5% (**) level. Similarly, asterisks in the bottom panel of columns 2 to 5 indicate significant differences between households in the bottom income quintile and households in other income quintiles.

Table 1 also presents a clear income gradient in proximity to public clinics with 20% of households in the poorest income quintile located more than 5 km from the nearest health facility as opposed to only 5% of households in the top income quintile (p < 0.01). Consistent with the income gradient in proximity to public clinics, higher-income households are more common in urban areas where facilities are closer, and high-income rural households tend to live closer to health facilities than low-income rural households.

Table 2 shows marked racial differences in health seeking behavior with around 60% of White and Coloured adults having consulted a health professional in the last year in contrast to only 44% of Black Africans (p < 0.01). Among those who had a health consultation, only 34% of Black Africans attended a private facility compared to 81% of Whites, which is partly due to substantially lower levels of health insurance (medical aid) coverage – less than one-tenth of Black Africans are covered.

**Table 2:** **Health seeking behavior and health characteristics among adults aged 18 years and older and children aged 5 years and under**

	**(1)**	**(2)**	**(3)**	**(4)**	**(5)**	**(6)**
	**Black African**		**Coloured**		**White**	
	**N**	**Mean**	**N**	**Mean**	**N**	**Mean**
**Adults aged 18 and older:**						
Health consultation in last year	9533	0.441	1838	0.621***	701	0.598***
Used private doctor or facility	4379	0.335	1143	0.464***	452	0.806***
Covered by medical insurance	9797	0.078	1896	0.161***	705	0.677***
Self-reports poor or fair health	9792	0.210	1901	0.219	709	0.130***
Obese (measured)	8543	0.262	1564	0.305***	494	0.344***
Measured hypertension	9053	0.323	1687	0.459***	540	0.443***
Aware of hypertension	3356	0.378	846	0.428**	276	0.526***
**Children aged 5 and under:**						
Skilled attendant at birth	2559	0.546	381	0.734***	63	0.981***

Authors’ calculation using NIDS and health facility data and post-stratification weights. The sample of adults aged 18 years and older excludes the small minority (8%) of individuals who moved recently (since 2006). The sample of children aged 5 years and younger excludes the small minority (10%) of children who have moved since they were born. Asterisks in columns 4 and 6 indicate whether Black African means are statistically significantly different from Coloured and White means respectively at the 1% (***) and 5% (**) level.

There are also substantial racial differences in both self-assessed and measured health status. White adults are 8 to 9 percentage points less likely than Black Africans and Coloureds to report being in fair or poor health (p < 0.01). This is despite Whites having higher prevalence of measured hypertension and obesity. Though White and Coloured adults have similar hypertension rates, Whites are 10 percentage points more likely to be aware of their hypertension than Coloureds (p < 0.01). Only 38% of hypertensive Black Africans are aware of their hypertension.

The bottom panel of Table 2 shows the percentage of children aged 5 and under who had a nurse or doctor present at their birth, a non-curative health service for which there is a universal need. Just over half (55%) of Black African children had a skilled birth attendant, which is statistically significantly lower than the 73% of Coloured children and 98% of White children (p < 0.01).

### Multivariate regression analysis

Table 3 uses multivariate regressions to examine the determinants of distance from the closest public health facility at the individual level for adults aged 18 and older. We examine both distance in kilometers and an indicator that the individual’s household is at least 2 kms from the nearest public clinic, a distance at which travel costs may start to matter. On average, Black Africans live 0.74 km further from the nearest clinic than Whites (p < 0.05). After controlling for education, household composition and income Black Africans are no longer significantly further away from the nearest clinic. In fact, point estimates are negative. Individuals in the lowest income quintile tend to be the furthest from public health facilities. The relationship between proximity to public clinics and education is complex, with those with tertiary education living 0.56 to 0.63 km further from the nearest clinic than individuals with less than primary education (p < 0.05). Households with more children and more pensioners tend to be located further away from clinics. Respondents who report themselves to be in poor or very poor health live on average 0.44 km closer to the nearest clinic than those individuals reporting better health. Results are substantively similar if we consider the likelihood of living at least 2 km from the nearest clinic rather than distance measured in kilometers.

**Table 3:** **Determinants of distance to the closest public clinic for adults aged 18 and older**

	**(1)**	**(2)**	**(3)**	**(4)**	**(5)**	**(6)**
**Outcome variable:**	**Distance (km) from nearest clinic**			**At least 2 km from nearest clinic**		
Black African	0.735**	−0.180	−0.185	0.041	−0.108	−0.115
	(0.310)	(0.311)	(0.323)	(0.087)	(0.072)	(0.074)
Coloured	−0.078	−0.613*	−0.601*	−0.206**	−0.284***	−0.292***
	(0.360)	(0.337)	(0.356)	(0.085)	(0.072)	(0.073)
Primary schooling		−0.161***	−0.156***		−0.026***	−0.024***
		(0.033)	(0.034)		(0.004)	(0.004)
Secondary schooling		−0.158***	−0.195***		−0.020***	−0.024***
		(0.051)	(0.056)		(0.007)	(0.007)
Completed high school		−0.028	−0.071		0.029	0.021
		(0.158)	(0.180)		(0.021)	(0.021)
Tertiary schooling		0.565**	0.630***		0.147*	0.171*
		(0.219)	(0.243)		(0.078)	(0.087)
2nd per capita income quintile		−0.484**	−0.376*		−0.085***	−0.065**
		(0.217)	(0.223)		(0.032)	(0.031)
3rd per capita income quintile		−0.582**	−0.480		−0.141***	−0.129***
		(0.295)	(0.315)		(0.039)	(0.040)
4th per capita income quintile		−0.755**	−0.710**		−0.193***	−0.193***
		(0.326)	(0.350)		(0.042)	(0.042)
5th per capita income quintile		−0.497	−0.499		−0.138***	−0.147***
		(0.341)	(0.370)		(0.050)	(0.051)
Number of children (<15) in household		0.186***	0.192***		0.030***	0.029***
		(0.052)	(0.057)		(0.008)	(0.008)
Number of individuals age 15 and older in household		−0.125**	−0.121**		−0.005	−0.005
		(0.056)	(0.054)		(0.011)	(0.010)
Number of pensioners in household		0.389**	0.408**		0.043	0.058**
		(0.164)	(0.177)		(0.028)	(0.029)
Self-reports poor or fair health			−0.441***			−0.055***
			(0.163)			(0.020)
Observations	14,968	14,843	12,400	14,968	14,843	12,400

Table reports regression coefficients from linear regressions. Sample excludes the small minority (8%) of individuals who moved recently (since 2006). The models in columns 1 to 6 include the set of control variables indicated in each column as well as an indicator that the individual is female and a quadratic in age.

Results are weighted using the post-stratification weights supplied by NIDS. Standard errors that allow for correlation in the unobservables between individuals from the same sampling cluster are presented in parentheses. ***p < 0.01, **p < 0.05, *p < 0.1

Table 4 uses multivariate regressions to examine distance decay in the likelihood of having a health consultation in the past year and to further investigate racial differences in health seeking behavior. Adults who are more than 2 km from the nearest health facility are 8 percentage points less likely to report a recent health consultation on average (p < 0.01). Controlling for gender, age and distance to the nearest facility, Black Africans are 9.5 percentage points less likely to report a recent health consultation (p < 0.01). The third column of Table 4 shows results where interaction terms between the indicator that the nearest public clinic is more than 2 km from the household and race were added to the regression. Although the interaction terms are statistically insignificant, the point estimates suggest that distance decay is greatest among Black South Africans. Adding controls for household composition, education level, a respondent self-reporting being in poor or fair health and household per capita income quintile only slightly reduces the Black African coefficient and it remains statistically significant. On average, individuals from households in the top two quintiles are around 7 to 10 percentage points more likely to have had a recent health consultation.

**Table 4:** **Determinants of having a health consultation in the past year for adults aged 18 and older**

	**(1)**	**(2)**	**(3)**	**(4)**	**(5)**
**Outcome variable:**	**Health consultation in the last year**				
More than 2 km from nearest clinic	−0.080***	−0.066***	−0.035	−0.065***	−0.050***
	(0.019)	(0.019)	(0.083)	(0.020)	(0.018)
African		−0.095***	−0.083**	−0.069	−0.084**
		(0.037)	(0.035)	(0.043)	(0.041)
Coloured		0.048	0.058	0.063	0.049
		(0.047)	(0.049)	(0.050)	(0.047)
African x more than 2 km			−0.035		
			(0.086)		
Coloured x more than 2 km			−0.028		
			(0.108)		
2nd per capita income quintile				0.030	0.018
				(0.019)	(0.018)
3rd per capita income quintile				0.017	0.015
				(0.023)	(0.022)
4th per capita income quintile				0.071***	0.085***
				(0.027)	(0.026)
5th per capita income quintile				0.101***	0.123***
				(0.035)	(0.036)
Self-reports poor or fair health					0.363***
					(0.017)
Observations	12,072	12,072	12,072	12,072	12,011

Table reports regression coefficients from linear regressions. Sample excludes the small minority (8%) of individuals who moved recently (since 2006). The models in columns 1 to 5 include the set of control variables indicated in each column. Regressions in columns 4 and 5 include indicators for primary, secondary, high school completion (matric) and post-matric, the number of individuals under the age of 15 in the household, the number of individuals aged 15 and older in the household and the number of individuals of pension eligible age. Results are weighted using the post-stratification weights supplied by NIDS. Standard errors that allow for correlation in the unobservables between individuals from the same sampling cluster are presented in parentheses. ***p < 0.01, **p < 0.05.

Table 5 shows that for children born within the last six years, the likelihood of having a skilled attendant at birth decreases with distance to the nearest clinic. Children in households more than 2 km from the nearest clinic are 8 percentage points less likely to have had a doctor or nurse present at their birth (p < 0.05). Controlling for proximity to public clinics, African and Coloured children are 42 and 25 percentage points less likely than White children to have a skilled attendant at birth respectively (p < 0.01). Results in the third column demonstrate racial differences in distance decay with only African children who live further from clinics being less likely to have had an attended birth. Including household composition variables and per capita income quintile in the regression roughly halves the African and Coloured coefficients although they remain substantial and statistically significant. With controls for income, distance is no longer significantly associated with having a skilled birth attendant.

**Table 5:** **Determinants of having a doctor or nurse present at birth for children aged 5 years and under**

	**(1)**	**(2)**	**(3)**	**(4)**
**Outcome variable:**	**Nurse or doctor present at birth**			
More than 2 km from nearest clinic	−0.082**	−0.059*	0.027	−0.025
	(0.034)	(0.033)	(0.023)	(0.033)
African		−0.424***	−0.394***	−0.226***
		(0.024)	(0.033)	(0.058)
Coloured		−0.253***	−0.231***	−0.107**
		(0.037)	(0.043)	(0.052)
African x more than 2 km			−0.091**	
			(0.044)	
Coloured x more than 2 km			−0.043	
			(0.095)	
2nd per capita income quintile				−0.046
				(0.035)
3rd per capita income quintile				−0.033
				(0.044)
4th per capita income quintile				0.108**
				(0.049)
5th per capita income quintile				0.186***
				(0.070)
Observations	3,003	3,003	3,003	3,003

Table reports regression coefficients from linear regressions. Sample excludes the small minority (10%) of children who have moved since they were born. The models in columns 1 to 4 include the set of control variables indicated in each column. The regression in column 4 also includes the number of individuals under the age of 15 in the household, the number of individuals aged 15 and older in the household and the number of individuals of pension eligible age in the household.

Results are weighted using the post-stratification weights supplied by NIDS. Standard errors that allow for correlation in the unobservables between individuals from the same sampling cluster are presented in parentheses. ***p < 0.01, **p < 0.05, *p < 0.1.

## Discussion

This study finds evidence that distance to health facilities poses a barrier for South Africans wishing to access health care. We use new data from the NIDS that enables us to calculate the distance from a respondent’s residence to nearby clinics. We find that many apartheid legacies remain in place. Ninety percent of South Africans live within 7 km of the nearest public clinic, and two-thirds live less than 2 km away. However, 14% of Black Africans live more than 5 km from the nearest facility, in contrast to only 8% of Coloureds and 4% of Whites, and they are substantially less likely to report a recent health consultation. Respondents in the poorest income quintiles are more likely to live further from the nearest health facility. Racial differentials persist in the likelihood of having a health consultation or an attended birth even after controlling for distance to the nearest facility, income quintile and household composition.

Travel costs (both monetary and time) constrain respondents’ choices about seeking health care. It is evident even when we control for health status in our regressions, or when we examine essential services like a birth attendant that even healthy South African women should access. Our results are consistent with previous findings that travel costs are but one of many barriers to accessing care, especially in remote and/or underserved areas. Notably, out-of-pocket costs [3,5], long queues [3,6], disrespectful treatment by facility staff [3,6], medication stock-outs [6] and perceived ineffective care [3,5] are tangible barriers that tend to be correlated with race, socio-economic status and rural–urban differentials [3]. Improvements in the quality of care provided in underserved areas have the potential to reduce racial differentials in access to and utilization of health care.

### Limitations

The NIDS data enables us to assess factors that the literature suggests to be important in mediating the role of distance in health care utilization. However, like most of the available literature we have to be cautious in interpreting these results as causal. Constrained choices about where individuals choose to live relative to health facilities may bias our estimates. An individual’s health status will influence his or her ability to earn income and, perniciously, individuals with poor health may have less choice about where they live relative to health clinics.

One limitation of our data is that it contains information only about the most recent health consultation rather than on all health utilization in a period of time. Types of consultations that occur more frequently, such as check-ups, will be over-represented in the data compared to consultations that occur less frequently, such as for acute illness. Our estimates of the relationship between distance to health facilities on utilization would be more precise if the NIDS data included information on the mode of transport used to travel to the clinic. Our data do not include estimates of expenditure on health care or travel to facilities so we are unable to assess this directly.

The scale up and decentralization of ART provision since 2008 is likely to have reduced the racial disparities evident in our study as HIV + individuals, who are much more likely to be Black African, are able to meet their health care needs at nearer clinics. However, distance disparities evident in 2008 may have long-lasting effects on health disparities.

## Conclusion

Our results have two clear policy implications. First, care should be taken to minimize the distance that poor South Africans must travel to obtain health care by situating health facilities in under-served areas and near places people travel for other purposes, such as work. A reduction in travel costs could lead to a substantial increase in health care utilization, especially for preventative and chronic care. Decentralization of services is one way to reduce travel time and ameliorate disparities, especially if the quality of care is maintained through the decentralization. Second, reducing the shadow price of using a service (i.e. the full opportunity cost; see [26]) by, for example, improving the quality of care [27], decreasing stigmatization of disease states [28], providing transportation vouchers or increasing the range of health services available at each health point will raise the benefit received per visit and increase the willingness to travel.

Distance plays a complex role in mediating health care utilization behavior, and more research is needed to characterize the relationship between the need for and utilization of health care. In particular, future studies should examine how the severity of the health concern and the perceived quality of care at health service points influence care-seeking decisions. Even when public health care is provided free of charge or on a sliding scale, monetary and time costs of travel present a salient barrier for economically vulnerable populations.
